# Age- and Organ-Specific Differences in Mitochondrial Bioenergetics in Brown Norway Rats

**DOI:** 10.1155/2020/7232614

**Published:** 2020-04-01

**Authors:** Jignesh D. Pandya, Matthew Valdez, Joyce E. Royland, Robert C. MacPhail, Patrick G. Sullivan, Prasada Rao S. Kodavanti

**Affiliations:** ^1^Spinal Cord and Brain Injury Research Center, Department of Neuroscience, University of Kentucky, Lexington, KY 40536, USA; ^2^Oak Ridge Institute for Science and Education, U.S. Department of Energy, Oak Ridge, TN 37831, USA; ^3^Neurological and Endocrine Toxicology Branch, Public Health and Integrated Toxicology Division, CPHEA/ORD, U.S. Environmental Protection Agency, Research Triangle Park, Durham, NC 27711, USA

## Abstract

Mitochondria play a central role in energy homeostasis and act as regulatory checkpoints for downstream metabolic responses and cell senescence processes during an entire life span. Acute or chronic environmental toxicant exposures have shown deleterious organ-specific human health issues at various life stages. Since mitochondria are a prime target for ensuing cellular bioenergetics responses and senescence, it is essential to understand mitochondrial bioenergetic responses in different organs over multiple life stages. Therefore, in the present study, we evaluated mitochondrial bioenergetic parameters in the liver, lung, and heart in four diverse age groups (young: 1 month; adult: 4 months; middle-aged: 12 months; old-aged: 24 month) using male Brown Norway rats as a model of aging (*n* = 5 sample size/organ/age group) and compared them with our previously published results on brain. Real-time mitochondrial bioenergetic parameters (i.e., State III, State IV, and State V) were measured using the Seahorse Extracellular Flux Analyzer. Additionally, mitochondrial enzyme pyruvate dehydrogenase complex (PDHC), Complex I, Complex II, and Complex IV activities were measured using Synergy HT plate reader. Our results indicated that nearly in all parameters, significant age- and organ-specific interactions were observed. We observed age-specific declines in State III (i.e., ATP synthesis rate) responses in both the heart and lung, where opposite was observed in the liver as age advances. Across the age, the heart has highest enzyme activities than the liver and lung. Interestingly, heart and liver mitochondrial bioenergetic rates and enzyme activities remain higher than the lung, which specifies their higher metabolic capabilities than the lung. Amongst all, bioenergetic rates and enzyme activities in the lung remain lowest suggesting the lung may display higher vulnerability and lower resilience to environmental toxicants during aging than other organs tested here. Overall, these age- and organ-specific findings may facilitate a more contextualized understanding of mitochondrial bioenergetic outcomes when considering the interactions of age-related sensitivities with exposure to chemical stressors from the environment.

## 1. Introduction

Mitochondria are known as the “powerhouse” of the cell. Metabolic breakdown of complex foods yields glucose, fatty acid, or amino acids via digestion, and then these components are further oxidatively metabolized by mitochondria in cells based on energy needs of an organ. Since glucose is the primary energy source of all organs, it is further metabolized to pyruvate in the cytosol via glycolysis. The pyruvate dehydrogenase complex (PDHC) enzyme is an entry point of the Krebs cycle where it feeds acetyl groups derived from pyruvate to coenzyme A, thereby initiating cyclic aerobic oxidation of substrates and generating reducing equivalents, i.e., NADH and FADH_2_. Both of them donate electrons to electron transport chain (ETC) Complex enzymes I and II, respectively. Electrons are then transported through Complexes III and IV to the Complex IV as final acceptor of O_2_ to generate water. During this aerobic respiration process, the flow of electrons is coupled to the transfer of protons from the mitochondrial matrix to the intermembrane space and establish the mitochondrial membrane potential (ΔΨ_m_), which is used by Complex V (i.e., ATP synthase) to generate energy in the form of adenosine triphosphate (ATP).

Although highly orchestrated and conserved, this ETC electron transfer process usually generates minimal electron leaks (∼1% of respiration) in healthy condition. This electron leakage targets oxygen and nitrogen molecules in the vicinity and may yield toxic products such as reactive oxygen/nitrogen species (ROS/RNS) [1, 2]. The cellular antioxidant system regulates these ROS/RNS levels. In recent years, ROS have been described in signaling pathways that are potentially beneficial to cells [3]; however, their overproduction is more commonly known to damage proteins, lipids, and DNA. Due to these complex and central cellular processes governed by mitochondria, several theories of aging have been discussed earlier. They are outlined based on rate-of-living theory: high metabolic rate = decreased life span [4]; mitochondrial decline theory: decreased mitochondrial function = decreased life span [5]; uncoupling-to-survive theory: increased uncoupling activity = increased life span [6]; mitohormesis: moderate, chronic ROS production = increased life span [7]; free radical theory: increased ROS = decreased life span [8]; and damage theory: accumulated damage from imperfect biomolecules = decreased lifespan [9].

The rate of reported acute and long-term exposures of toxic drugs and pollutants on mitochondria function has increased over recent decades [10, 11]. In fact, mitochondrial dysfunctions are some of the most predictable findings across high priority chemicals screening assays performed at the National Toxicology Program [12]. While mitotoxicants such as pentachlorophenol [13], carbon monoxide [14], rotenone [15], and oligomycin [16] have been known for decades, newer chemicals such as pesticides, herbicides, cigarette and cookstove smoke, and even air pollution have all been found to impact mitochondria function [17]. For example, exposure to trichloroethylene, an environmental toxicant used in producing refrigerants and to remove grease from metal products, produces mitochondrial complex I dysfunction which is observed in a neurological disorder, i.e., both Parkinsonism patients and a rat model [18, 19]. In addition to toxicant interactions, mitochondria are also central to other pathologies. Mitochondrial dysfunction has been implicated in the etiology of heart failure [20]. Specific patterns of irregular mitochondrial signatures are indicators of several lung disease such as asthma, chronic obstructive pulmonary disease, and even cancer [21]. Within the liver, the cause of nonalcoholic fatty liver disease is unknown, but the prevailing theories suggest that decreased capacity of mitochondria to oxidize fatty acids may be involved in the onset of the disease [22].

Given the role of mitochondria in both aging and toxicity, it is essential to characterize mitochondrial bioenergetics across all life stages in order to fully understand toxicological adverse outcome pathways. In addition, effects of mitochondrial dysfunction have empirically been found to be cell- and tissue-specific [10] which complicate the matter further. In this report, we present an in-depth, comprehensive study to advance our understanding of the role of mitochondrial bioenergetic function in aging. The study was carried out in the Brown Norway rat, a model of healthy aging. Mitochondrial bioenergetics and enzyme activities were evaluated at multiple ages from young, adult, middle-aged, and old animals (1, 4, 12, and 24 months, respectively) in different organs (heart, liver, and lung) and compared with our previously published data in the brain using frontal cortex as a representative [23]. Mitochondrial respiration parameters State III rate (ATP synthesis capacity), State IV rate (proton leakage), and State V rate (maximal electron transport) were measured together with mitochondrial enzyme (PDHC and Complexes I, II, and IV) activities. Taken together, these parameters reveal the overall organ-specific mitochondrial bioenergetic efficiency across lifespan. The comprehensive design of this study will provide beneficial characterization of age-related metabolic status changes in individual organs for future studies to assess the adverse effects of mitotoxicants (environmental chemicals) at different life stages. Additionally, knowledge gained in this study will aid in the understanding of age-induced sensitivity towards environmental chemicals.

## 2. Materials and Methods

### 2.1. Animals

Male Brown Norway rats from four different life stages were examined (i.e., 1 month (1M): young, 4 months (4M): adult, 12 months (12M): middle-aged, and 24 months (24M): old-aged) in this study. Animals (*N* = 5-6 rats/group) were purchased from the National Institutes of Health (NIA) animal facilities at their appropriate age, less than 2 weeks prior to the actual experiment. All animal protocol and procedures were approved by the University of Kentucky Institutional Animal Care and Use Committee. Animals were maintained at University of Kentucky animal facilities on a regular 12-hour light/12-hour dark cycle in temperature-controlled rooms (25°C) for the first week to acclimate to the environment. Animals were pair-housed and provided with ad lib rat chow and water. To limit age variability within groups, all animals were used within 3 days for the 1M age group and to a maximum of 14 days for the 4M, 12M, and 24M age groups. After the 1-week acclimatization, all animals were randomly assigned dates of sacrifice. Two animals per day (except for 1M animals where 2 animals were pooled per single sample giving 4 animals/day) were processed for organ-specific mitochondrial isolation and bioenergetic parameter measurements.

### 2.2. Isolation of Mitochondria

Mitochondria from 3 visceral organs (i.e., heart, liver, and lung) were isolated together under identical experimental conditions on each experimental day using an established and previously published ficoll-based mitochondrial ultrapurification procedure [24–27]. Mitochondria from five brain regions were also isolated at the same time from the same cohort of animals and the results were published earlier [23]. Throughout the mitochondrial isolation period, samples were kept at 4°C on ice, unless otherwise specified. Animals were asphyxiated with CO_2_ and rapidly decapitated. Following decapitation, the heart, liver, and lung were rapidly removed and placed in a beaker of ice-cold (4°C) mitochondrial isolation buffer (MIB) composed of 215 mM Mannitol, 75 mM sucrose, 0.1% BSA, 1 mM EGTA, and 20 mM HEPES at pH 7.2. All organs of interest were rapidly dissected apart, placed in prelabeled 5 ml tubes containing 1 ml of MIB buffer, and homogenized. Tissue homogenates were centrifuged at 1300 ×g for 3 min to remove cell debris and nuclei, and the collected supernatant was then centrifuged at 13,000 ×g for 10 min to pellet the mitochondria. The resultant crude mitochondrial pellet was then resuspended in 500 *μ*l of MIB and placed on top of discontinuous ficoll gradient (layered 2 ml of 7.5% ficoll solution on top of 2 ml of 10% ficoll solution) and centrifuged at 100,000 ×g for 30 min using a Beckman ultracentrifuge with SW55Ti rotor (Beckman Coulter, IN, USA). Ultrapure mitochondrial pellets formed at the bottom were carefully removed and were resuspended in 2 ml MIB (without 1 mM EGTA buffer) and centrifuged at 10,000 ×g for 10 min. The washed mitochondrial pellets were finally resuspended in MIB^−^ to a final concentration of ∼10 *μ*g/*μ*l mitochondrial protein (volume varied depending on organs). Mitochondrial protein concentration was determined using the BCA protein assay kit measuring absorbance at 562 nm with BioTek Synergy HT plate reader (Winooski, VT, USA).

### 2.3. Mitochondrial Bioenergetics Measurements

Measurements of mitochondrial bioenergetics in isolated mitochondrial samples were performed immediately after isolation using a slightly modified published protocol of Seahorse XF24 Extracellular Flux Analyzer (Agilent Inc., Santa Clara, CA) [28–31].In brief, stock solutions of mitochondrial substrates of 500 mM pyruvate, 250 mM malate, and 30 mM ADP plus assay solutions of 1 mg/ml oligomycin-A, 1 mM carbonyl cyanide-4 (trifluoromethoxy) phenylhydrazone (FCCP), 1 mM rotenone, and 1 M succinate were prepared. The pH was adjusted to 7.2, and the solutions were stored at −20°C. The day before the planned experiment, a 24 well dual-analyte sensor cartridge was hydrated and kept in a non-CO_2_ incubator at 37°C. On the scheduled day of the experiment, the sensor cartridge ports A to D were loaded with the appropriate mitochondrial substrates or inhibitors (freshly prepared 10X working concentration made from stocks) and sequentially injected into the assay plate according to the protocol procedure to reach a final concentration of the compound (1X) in each well. All mitochondrial working stocks were prepared in respiration buffer (RB) composed of 215 mM mannitol, 75 mM sucrose, 0.1% BSA, 20 mM HEPES, 2 mM MgCl_2_, and 2.5 mM KH_2_PO_4_ at pH 7.2 and stored at 4°C. The amount of substrates/inhibitors loaded for each port is based upon the initial 500 *μ*l RB volume in the mitochondrial plate as follows: Port A: 50 *μ*l (mixture of pyruvate, malate, and ADP), Port B: 55 *μ*l (oligomycin A), Port C: 60 *μ*l (FCCP), and Port D: 65 *μ*l (rotenone and succinate). Once the sensor cartridge was loaded with all of the experimental reagents, it was placed into the Seahorse XF24 Flux Analyzer for automated calibration.

Seahorse Standard XF24 assay plates were utilized for mitochondrial analysis. Mitochondria (15 *μ*g) from three visceral organs were analyzed together on a single plate. Purified mitochondria as described above were added to RB buffer to give a final concentration of 15 *μ*g mitochondrial protein in 50 *μ*l RB and added to experimental wells. Background control wells contained 50 *μ*l of RB without mitochondria. Loaded XF24 plates were centrifuged at 3,000 rpm for 4 minutes at room temperature in a tabletop centrifuge to attach mitochondria to the well bottom. Following centrifugation, 450 *μ*l (37°C) of prewarmed RB was gently added to each well for a final volume of 500 *μ*l per well. Following sensor cartridge calibration, plates were placed into the Seahorse XF24 Extracellular Flux analyzer for mitochondrial bioenergetics analysis.

An optimized protocol was utilized for the analysis of bioenergetics function in purified mitochondria using the Seahorse Biosciences XF24 Flux Analyzer. The protocol contains cyclic steps of a cartridge probe calibration, baseline readings, sequential injections of substrates/inhibitors via ports A thru D, substrates mixing in the assay system, a delay for well O_2_ equilibration, and then measurement of the oxygen consumption rates (OCR) as elaborated upon previously [28, 30, 31]. The OCR rates of State III responses in presence of 5 mM pyruvate, 2.5 mM malate, and 1 mM ADP (Port A) were measured followed by State IV response in presence of 1 *μ*M oligomycin A (Port B). Sequentially, State V_FCCP_ and State V_Succ_ OCR rates were recorded automatically in presence of 4 *μ*M carbonyl cyanide 4-(trifluoromethoxy)phenyl hydrazone (i.e., FCCP) (Port C) and 0.1 *μ*M rotenone plus 10 mM of succinate (Port D), respectively. After experiment, the Seahorse file was configured to display OCR rates for Ports A–D in a point-to-point mode. The highest OCR rates for ports A–D were used to perform data analysis. Data are reported as OCR for the various brain regions isolated from the four age groups.

### 2.4. Mitochondrial Enzyme Activity Assessments

Mitochondrial Krebs cycle gatekeeper and electron transport chain (ETC) enzyme activities were performed with an automated 96-well microplate reader (Bio-Tek Instruments Inc., Winooski, VT) as described in previously published articles [32–34]. For all enzyme assays, stored mitochondrial samples were diluted to 1 *μ*g/*μ*l in 10 mM KH_2_PO_4_ followed by freeze-thaw and sonication together three times to release enzymes before measuring enzyme activities for all samples on single plate at 37°C.

The mitochondrial Krebs cycle gatekeeper enzyme pyruvate dehydrogenase complex (PDHC) enzyme activity was measured by the production of NADH as previously described [32, 33], with slight modifications. Ficoll-purified mitochondrial proteins (20 *μ*g/well) were added in triplicate to wells containing 100 *μ*l total assay volume of reaction buffer (50 mM KCl, 10 mM HEPES pH 7.4, 0.3 mM thiamine pyrophosphate, 10 *μ*M CaCl_2_, 0.2 mM MgCl_2_, 5 mM pyruvate, 1 *μ*M rotenone, and 0.2 mM NAD^+^) in a 96-well format. The enzyme reaction was started by addition of 0.14 mM Coenzyme A/well. Measurements were taken at 1 min intervals for 3 min at Excitation/Emission (Ex *λ* 340 nm/Em *λ* 460 nm). The PDHC activity was calculated and expressed as nmol/min/mg mitochondrial protein.

The Complex I (NADH dehydrogenase) enzyme activity was performed in triplicate in a reaction buffer of 25 mM KH_2_PO_4_ (pH 7.2) containing 5 mM MgCl_2_, 1 mM KCN, 1 mg/ml BSA, and 150 *μ*M NADH. Mitochondrial proteins (20 *μ*g) were added to the reaction buffer in 100 *μ*l of assay volume, and the assay was performed in the presence or absence of rotenone (10 *μ*M) to determine the rotenone-insensitive and the rotenone-sensitive Complex I enzyme activity. The rotenone-insensitive rate of NADH oxidation was subtracted to obtain rotenone-sensitive activity. The reaction was started by the addition of 50 *μ*M of coenzyme Q1. The disappearance of NADH over time was measured at 1 min intervals for 20 min. The assay was performed at Ex *λ* 340 nm/Em *λ* 460 nm. Enzyme activity was calculated and expressed as nmol/min/mg mitochondrial protein.

Mitochondrial Complex II (succinate dehydrogenase) enzyme activity was determined by measuring the change in absorbance of a dye, 2,6-Dichlorophenol Indophenol (DCIP), at 600 nm using a BioTek Synergy HT plate reader (BioTek, Winooski, VT). In brief, 20 *μ*g of mitochondrial protein was added to 200 mM KH_2_PO_4_ pH 7.0 assay buffer containing 20 mM K-Succinate, 10 *μ*M EDTA, 0.01% Tx-100, and 1 *μ*g coenzyme Q10 in a total assay volume of 100 *μ*l/well in triplicate. An approximately 20 mg% DCIP dye was added to obtain initial absorbance readings between 0.8–1.0 at 600 nm. The decrease in absorbance was measured constantly for 15 min and the activity was expressed as in terms of nmols of succinate oxidized/min/mg mitochondrial protein [25, 35].

The mitochondrial Complex IV (cytochrome c oxidase) activity was measured in 10 mM KH_2_PO_4_ buffer (pH 7.4) coincubated with 20 *μ*g mitochondrial protein in triplicate. The reaction was initiated by adding 50 *μ*M reduced cytochrome c (final total assay volume of 100 *μ*l). The rate of oxidation of cytochrome c was measured as the decreased absorbance of reduced cytochrome c at 550 nm for 2 min. The rate constant (K) of oxidation of cytochrome c was calculated and expressed as K/min/mg mitochondrial protein [28, 36].

### 2.5. Statistical Analysis

**Figure 1:**
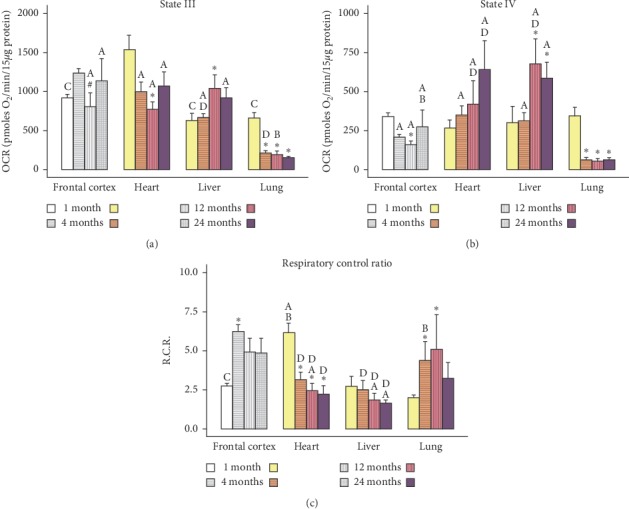
Age-dependent changes in State III (a) and State IV respiration (b) rates and respiratory control ratio (c) in different organs. Values are expressed as means ± SEM from *n* = 5-6 observations/group. The differences between pairwise comparisons were considered statistically significant at *p* < 0.05. ^*∗*^Significantly different from 1M rats; ^#^significantly different from 4M rats; ^A^significantly different from lung; ^B^significantly different from liver; ^C^significantly different from heart; ^D^significantly different from frontal cortex; SEM, standard error of the mean. Grey-scale textured bars represent frontal cortex data from our previous report (adapted from Pandya et al. [23], with permission).

**Figure 2:**
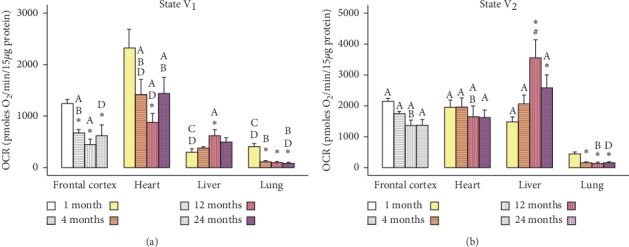
Age-dependent changes in State V_1_ (a) and State V_2_ OCR rates (b) in different organs. Values are expressed as means ± SEM from *n* = 5-6 observations/group. The differences between pairwise comparisons were considered statistically significant at *p* < 0.05. ^*∗*^Significantly different from 1M rats; ^A^significantly different from lung; ^B^significantly different from liver; ^C^significantly different from heart; ^D^significantly different from frontal cortex; SEM, standard error of the mean. Grey-scale bars represent frontal cortex data from our previous report (adapted from Pandya et al. [23], with permission).

**Figure 3:**
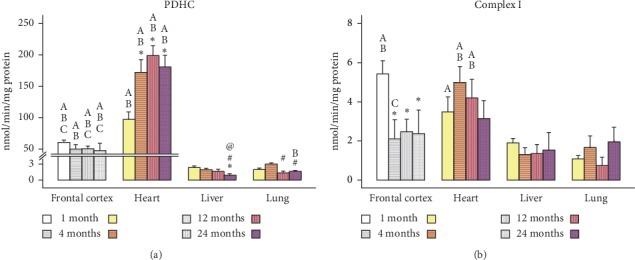
Age-dependent changes in activities of PDHC (a) and Complex I (b) enzymes in different organs. Values are expressed as means ± SEM from *n* = 5 observations/group. The differences between pairwise comparisons were considered statistically significant at *p* < 0.05. ^*∗*^Significantly different from 1M rats; ^#^significantly different from 4M rats; ^@^significantly different from 12M rats; ^A^significantly different from lung; ^B^significantly different from liver; ^C^significantly different from heart; SEM, standard error of the mean. Grey-scale bars represent frontal cortex data from our previous report (adapted from Pandya et al. [23], with permission). Note the *y*-axis scale difference between frontal cortex and heart versus liver and lung.

**Figure 4:**
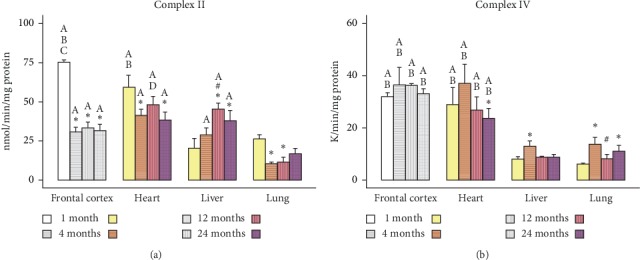
Age-dependent changes in activities of Complex II (a) and Complex IV (b) enzymes in different organs. Values are expressed as means ± SEM from *n* = 5 observations/group. The differences between pairwise comparisons were considered statistically significant at *p* < 0.05. ^*∗*^Significantly different from 1M rats; ^#^significantly different from 4M rats; ^A^significantly different from lung; ^B^significantly different from liver; ^C^significantly different from heart; ^D^significantly different from frontal cortex; SEM, standard error of the mean. Grey-scale bars represent frontal cortex data from our previous report (adapted from Pandya et al. [23], with permission).

The values represented in Figures1–4 are mean ± SEM (*n* = 5-6 sample size/organ/age group). Statistical analysis was performed using RStudio [37]. Raw data were imported and organized and column statistics were generated with the dplyr package [38]. Linear models were constructed using the lm() function in the R base package. Models were defined as the main effects and interaction of age and organ for each parameter. Normality and homogeneity of variances assumptions were tested with Shapiro–Wilk test and Levene's test, respectively. When subsets of data did not satisfy normality and homogeneity of variance assumptions, the linear models were modified by log-transforming the data. All subsets of data, except Complex I and II, were log-transformed.

All linear models were analyzed via two-way analysis of variance. Significant main effects and interactions were followed with a post hoc multiple comparisons test. Pairwise comparisons of age and organ groups were performed using Fisher's least significant difference post hoc test. Statistical significance was defined at *p* < 0.05 for all determinations. All figures were generated using the ggplot2 [39] and cowplot [40] packages.

## 3. Results

**Figure 5:**
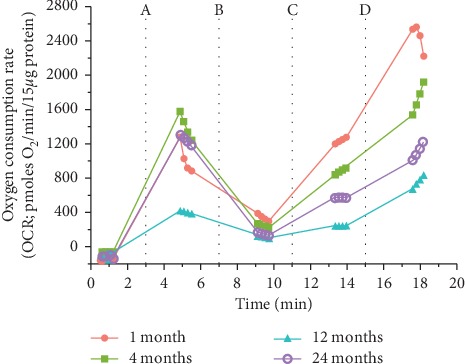
Typical traces of brain (frontal cortex) mitochondrial bioenergetics using Seahorse Extracellular Flux Analyzer from rats that are 1 month, 4 months, 12 months, and 24 months of age. Each line shown is representative point-to-point display of patterns of OCR respiration rates at 1, 4, 12, or 24 months of age. Oxygen consumption rates (OCR) were measured with addition of substrates/inhibitors of electron transport chain. Vertical lines represent addition of compounds used to determine the various respiration states: (A) substrates (pyruvate, malate, and adenosine diphosphate) for State III; (B) oligomycin for State IV; (C) carbonyl cyanide 4-(trifluoromethoxy)phenylhydrazone (FCCP) for State V_1_; and (D) succinate and rotenone for State V_2_.

In the present study, we used the Seahorse Extracellular Flux Analyzer (Seahorse Bioscience XF-24) to analyze bioenergetics in 3 visceral organs and compared them with frontal cortex (representative of brain) in four different life stages (young: 1M; adult: 4M; middle-aged: 12M; and old aged: 24M) of Brown Norway rats. A typical pattern of oxygen consumption rate (OCR, a measure of oxidative phosphorylation) of brain mitochondria illustrates age-specific changes in mitochondrial respiration rates as shown in Figure 5. All other figures (Figure 1–4) depict average activities from 5-6 biological replicates by organ across age. Mitochondrial bioenergetic parameters included were State III respiration rate (ATP synthesis capacity), State IV respiration rate (proton leaking), and State V respiration rates (maximal electron transport) in presence of Complex I or Complex II substrates (Figures 1 and 2). Additionally, we measured mitochondrial PDHC, Complex I, Complex II, and Complex IV enzyme activities to observe comprehensive picture of differences in bioenergetic profile following different life stages preclinically (Figures 3 and 4).

### 3.1. Age- and Organ-specific Differences in Mitochondrial Bioenergetics

Age- and organ-specific differences in mitochondrial bioenergetics were present in nearly all organs. Two-way ANOVA analysis of State III respiration rate (Figure 1(a)), which is related to ATP synthesis rate, indicated a significant interaction of age and organ (*F*_9,68_ = 5.822, *p*=5.612*e*^−6^) throughout life stages, with all organs displaying higher average respiration rates compared to lung as determined by *post hoc* pairwise comparisons. These differences appear to arise after 1 month in the lung, with all State III respirations rates dropped near to 200 OCR as the animal aged. There was also a trend of decreasing State III respiration in the heart, with the largest change observed between 1M and 12M. Interestingly, this decreased state III respiration rate in the heart rebounded after 24M. State III respiration remains consistent between 1M to 4M in the liver with a significant increase noted at 12M and 24M.

State IV respiration rate (Figure 1(b)), which represents proton leakage through the mitochondrial membrane, also had a significant interaction of age and organ as indicated by two-way ANOVA (*F*_9,68_ = 4.987, *p* = 3.743*e*^−5^). State IV respiration in the heart steadily increased over time, but this trend was not significant may be due to higher standard errors in experiments within group due to limited sample size used in the study. Matching the pattern of State III respiration in the liver, State IV also displayed a significant increase at 12M that persisted until 24M. Just as in State III respiration, the lung had an overall lower average State IV respiratory rates compared to all other organs. Also consistent with the State III respiration in the lung, proton leakage decreased after 1M similarly across all ages. Again, when compared organ-specific effects of State IV across age, we observed similar pattern of State IV as we have seen in state III respiration rates. More specifically, lung mitochondrial State IV rates were comparable with heart and liver in 1M. It is interesting to note that, as the heart and liver are the major metabolic organs of the body, their mitochondria showed higher State III and State IV respiration rates compared tothe lung, which has the least respiratory capacity at 1M age. As age increases, the 4M-24M group of lung mitochondria showed significantly less proton leakage than the heart and liver.

We measured mitochondrial State V respiration rate (maximal electron transport) by applying an uncoupling agent (FCCP) through Port C that triggers collapse of the proton gradients across mitochondrial inner membrane and disrupts the mitochondrial membrane potential in combination with either a Complex I or Complex II specific substrate addition (Figure 2). The State V_1_ (Figure 2(a)) measures the uncoupling rates in the presence of the Complex 1 substrates pyruvate and malate. The State V_2_ (Figure 2(b)) measures the uncoupling rates in the presence of the Complex II substrate succinate and the Complex I inhibitor, and rotenone was added through port D to block complex I driven respiration. These OCR rates measure maximal respiratory capacity of ETC through Complex I or Complex II, since ETC respiration is delinked with oxidative phosphorylation using ATP synthase inhibitor oligomycin in the Seahorse assay chamber. Like the other parameters, two-way ANOVA indicated a significant interaction of age and organ for State V_1_ respiration (*F*_3,68_ = 4.531; *p* = 0.0001) (Figure 2(a)). There was a trend of decreasing State V_1_ respiration in the heart, with a significant difference between 1M and 12M, whereas at 24M, the rate increased. Conversely, State V_1_ respiration in the liver increased with age with a significant difference between 1M and 12M. Then again, in the lung after 1M, there was a significant decrease in State V_1_ respiration that persisted until 24M. Lung State V_1_ respiration followed similar pattern as state III respiration. The maximal respiratory capacity was significantly declined at later age between 4M–24 M as compared to 1M age group. When compared organ specificity, in young 1M group, the heart State V_1_ rates were higher than both liver and lung. Subsequently, between 4M–24 M period, the heart State V_1_ rates remain highest than moderately respired liver, followed by the least respired lung.

Like State V_1_, two-way ANOVA revealed a significant interaction of age and organ (*F*_9,68_ = 5.104, *p*=2.855*e*^−5^) in State V_2_ (Figure 2(b)), Complex II driven respiration. In the heart, there was an apparent decrease in State V_2_ respiration from 12M to 24M that did not reach significance. There was a significant increase in State V_2_ from 1M to 12M in the liver. In the lung, just as in all other mitochondrial bioenergetic parameters, there was a significant decrease in State V_2_ respiration after 1M across all other ages. It is interesting to note that the State V_2_ activity remained identical in heart across age group.

### 3.2. Age- and Organ-Specific Differences in Mitochondrial Enzymes

PDHC is the key regulatory enzyme of cellular metabolism because it links the Krebs cycle and subsequent oxidative phosphorylation with glycolysis and gluconeogenesis. In addition to mitochondrial respiratory parameters, we also analyzed PDHC enzyme activity (Figure 3(a)) in three visceral organs across life stages and compared them with the frontal cortex. Two-way ANOVA analysis indicated a significant interaction of age and organ on PDHC enzyme activity (*F*_9,24_ = 3.881; *p* = 0.0005). The differences driving this effect appeared to be the high PDHC activity in the heart and the much lower activities across both the liver and lung. In the heart, which had the highest levels of PDHC activity, there was significantly increased and sustained enzymatic activity after 1M. In the liver, there was a trend of decreasing PDHC enzyme activity with significant differences between 1M to 24M and 4M to 24M. In the lung, PDHC activity remained higher from 1M to 4M and then there was a significant drop at 12M and 24M.

Mitochondrial Complex I (Figure 3(b)), which is the primary entry point to the ETC, varied across organ with a significant main effect of organ (*F*_3,64_ = 11.74, *p*=3.19*e*^−6^). The highest Complex I enzyme activities were found in the heart, similar to PDHC activity. Within all other organ groups, there were no significant effects of age; however, there was a trend consistent with other measures of mitochondrial bioenergetics. For instance, analogous to State V_1_ and PDHC, the Complex I activities in the lung and liver were considerably less than in heart.

Mitochondrial Complex II (Figure 4(a)), which is a secondary entry point to the ETC as well as a component of the Krebs cycle, was variable across organs. Two-way ANOVA indicated a significant interaction of age and organ in Complex II activity (*F*_9,64_ = 8.257; *p*=4.915*e*^−8^). Post hoc multiple comparisons indicated that all Complex II enzyme activity in the lung was significantly less compared to nearly all other organs. Within each organ, the effect of age was nearly identical to that of State V_2_ (Figure 2(b)) or Complex II driven maximal respiration. In the heart, there was a significant trend of decreasing Complex II activity after 1M. In the liver, there was a trend of increasing Complex II activity from 1M to 12M where the difference reached significance and plateau throughout 24M. This effect was also captured in the State V_2_ respiration measures within the liver. In the lung, Complex II activity was highest at 1M and then significantly decreased thereafter in 4M and 12M.

Mitochondrial Complex IV (Figure 4(b)), the last enzyme in the ETC, was also variable across organs, and two-way ANOVA analysis found significant main effects of age (*F*_3,64_ = 4.95; *p* = 0.0037) and organ (*F*_3,64_ = 95.1; *p* = 1.523*e*^−23^). Post hoc multiple comparisons indicated that all Complex IV enzyme activity in the lung and liver was significantly less compared to the heart. In the heart, there was a bell-shaped trend of Complex IV activity across age, where 4M showed higher activity than other age groups. In the liver, there was a significant transient increase in Complex IV from 1M to 4M, with a return to 1M activity levels from 12M to 24M. In the lung, there was a significant and sustained increase in Complex IV activity after 1M.

## 4. Discussion

Aging is a multifactorial process thought to be influenced by internal factors (e.g., genetics, stress, and nutrition) and external factors (e.g., lifestyle and environmental toxicants). There are several theories regarding the mechanism of aging which, based on their core assumptions, can be grouped into two categories: (1) aging is programmed or (2) aging is caused by an accumulation of damage [41]. Over the decades, there has been an increase in the understanding of cause vs. effect relationship in aging. In its inception, the free radical theory of aging is specifically limited to oxidative damage imparted by free radicals. The next evolution of that idea, the mitochondrial theory of aging, expanded the scope of damage to include any source of mitochondrial dysfunction such as increased mitochondrial electron leakage, mtDNA mutations, oxidative stress, or breakdown in the membrane potential to underline the cause of aging. More recently, it has been postulated [9] that mitochondrial bioenergetic dysfunction may be only one of many types of damage that accumulate from the imperfectness of biological systems over time. Other factors such as protein turnover rates may also contribute to the overall cellular bioenergetics capacity, thereby influencing the aging process [42]. Whichever doctrine one adheres to, it is undeniable that age-dependent decline in mitochondrial dysfunction does occur and does contribute to senescence. Whether or not mitochondrial dysfunction is the driving force of aging, the fact that environmental toxicants commonly interact with mitochondrial processes imposes an added threat to aging individuals. Current study was designed to extend our understanding based on current hypothesis that different organs may have altered metabolic energy demand and susceptibility/resilience to toxic chemicals within or across ages and therefore may contribute differently during altered life stages. To test whether energy metabolism differs in different organs and to investigate whether that metabolism changes over life span, we have examined mitochondrial function at various ages in a variety of organs associated with specific age-related deficits using a rat model of aging. Overall, current data indicated heart as the highly active metabolic organ followed by liver, and lung remained as the most vulnerable organ when considering the interactions of age-related sensitivities with exposure to chemical stressors from the environment perspective. Ultimately, these data may serve as a basic platform to test various stressors or toxicant effects on age- and organ-specific loss of homeostatic function.

In our previous study [23], we sought to characterize mitochondrial bioenergetic changes over time in various brain regions using an identical experimental condition in the identical age groups using Brown Norway rats. We found brain region-specific changes in the brain mitochondrial bioenergetics function over life stages. Given the susceptibility of mitochondria to environmental toxicants, we found these outcomes provided essential contextual information in the understanding of brain region susceptibility to environmental chemicals/toxicants during aging. This current study was designed to expand our understanding by characterizing visceral organ-specific mitochondrial bioenergetics at various life stages. We picked three visceral organs (i.e., heart, liver, and lung) which are considered to have higher metabolic capacities and critical targets to environmental chemicals following acute or chronic exposures. Based on current experimental data, we may better understand the metabolic capacity of these organs over age. This study may serve as a platform to screen various chemicals/toxic compounds based on their effects on mitochondrial respiration and metabolism over time. Additionally, these mitochondrial targets may be useful to delay progression of metabolic dysregulation across various life stages.

Mitochondrial bioenergetics and complex enzyme activities were studied in different organs and age groups using identical procedures. As described above, we found that for many mitochondrial parameters, age and organ interact to produce varying patterns of respiration and enzyme activities. The general pattern in the mitochondrial respiratory parameters (State III, IV, V_1_, and V_2_ respiration rates) across organ and over lifespan suggest that respiration is typically greatest in younger animals (1M) and then decreases over time until old age (24M). This pattern was not evident in all cases such as in the liver, where young animals (1M) had the lowest respiration rates of all parameters (State III, IV, V_1_, and V_2_) and middle-aged (12M) or old animals (24M) had the highest levels of respiration. This may be related to the development of that particular organ.

An interesting deviation from the general pattern of respiration can be seen in rates of State III and State V_1_ in the heart where the usual decline in respiration only lasted until middle-age (12M) but was then followed by an apparent rebound effect in old animals (24M). A similar pattern was seen previously in frontal cortex in both State IV and State V_1_ respiration rates [23]. Together both increasing State III (related to ATP synthesis) and State V_1_ (Complex I driven maximum electron transport rates) represent increasing Complex I substrate driven ATP synthesis rates. In further support of there being an increase in Complex I driven State III respiration in the heart is the increase in PDHC activity throughout the life of the animal. An increase in PDHC activity indicates more Complex I substrates could be available to compensate decreased ATP synthesis rates between 1M to 12M period. As previously shown, PDHC activity in the frontal cortex remained stable throughout the lifetime of the animal. Consistent with the mitochondrial theory of aging, it is not surprising to see a decline of ATP synthesis capacity with age in the heart which could indicate less efficient mitochondria over time. However, the rebound in State III rates is quite surprising which suggests a possible compensatory mechanism operated at old age specifically in the heart and frontal cortex. To our knowledge, no such compensatory mechanism as described above has been reported in the literature. This compensation could be a way for the body to prioritize specific organs energy requirements during the onset of senescence. We find this prioritization scheme likely as we show a similar trend in respiration rates (frontal cortex ∼ heart > liver > lung) in each vital organ across age groups.

In the liver, we found a decreasing respiratory control ratio over lifetime. The higher State III rates in the liver indicated increased metabolic demand starting in middle-age (12M). Meanwhile, some of the highest levels of proton leakage were detected in the liver at the same age (12M) and suggest in stark increase in uncoupling compared to younger animals. Furthermore, liver samples starting at 12M had the highest rates of Complex II driven uncoupling and significantly higher Complex II enzyme activity. This profile could be interpreted as reactive instead of proactive. Similar to this line, Serviddio et al. have previously reported increased uncoupling and proton leak in liver mitochondrial samples with aging, together with increased Complex I driven State III and State IV rates in liver in aged samples as compared to young group [43]. Moreover, cellular senescence drives age-dependent hepatic steatosis (fatty liver) [44], and the increase in free fatty acids has been shown to increase expression of certain uncoupling proteins in mitochondria [45]. Therefore, it is possible that the increase in leakage that is driving the age-related decrease in respiratory control may be related to the onset of fatty liver disease as described by others. In the present study, we did not assess fatty liver status. Future studies should assess liver mitochondrial uncoupling more in depth by activation with free fatty acids.

Activation of PDHC promotes glucose utilization for energy production, whereas inactivation conserves glucose. In the aging heart, it has been demonstrated that increased glucose utilization, via transgenic overexpression of glucose transporters in cardiomyocytes, produces a favorable metabolic phenotype that provides protections against ischemia [46]. In the heart (Figure 3(a)), there is a profound increase in glucose utilization as the animal ages. Perhaps, as suggested by Luptak et al., there exists a mechanism in the heart whereby increased glucose consumption can tender some protection against age-related complications. However, there is no further evidence of protection; in fact, there is quite the opposite. As glucose metabolism increases in the heart both ATP synthesis (State III) and Complex I driven maximal electron transport (State V_1_) decrease with age suggesting a breakdown in the transfer of electrons though the ETC. The higher PDHC activity may be a compensatory mechanism as we saw decreased ATP synthesis (state III respiration) with aging. Heart PDHC may have to overwork to compensate for age related energy loss. This aging-related breakdown of the ETC is further supported by the drastic increase in proton leakage (State IV) in the heart though life span. Whether this phenotype is suggestive of age-related heart dysfunction is not known.

## 5. Conclusions

In summary, we report differential age- and organ-specific changes in the Brown Norway rat mitochondrial function. In combination with our previous report [23] the brain region-, age-, and organ-specific patterns reported in our studies may serve as a guideline for assessing the relative vulnerability of specific organs to environmental chemical insult across life span.

## Data Availability

The data used to support the findings of the study are available from the corresponding author upon request.
